# A rapid and efficient method for the extraction and identification of menaquinones from *Actinomycetes* in wet biomass

**DOI:** 10.1186/s12866-021-02240-z

**Published:** 2021-06-09

**Authors:** Fuquan Xie, Shengxiang Pei, Xihuang Lin, Yun Tian, Gaiyun Zhang

**Affiliations:** 1 Key Laboratory of the Ministry of Education for Coastal and Wetland Ecosystems, School of Life Sciences, Xiamen University, Xiamen, 361102 Fujian PR China; 2 Key Laboratory of Marine Biogenetic Resources, Third Institute of Oceanography, Ministry of Natural Resources, Xiamen, 361005 Fujian PR China; 3 Analysis and test center, Third Institute of Oceanography, Ministry of Natural Resources, Xiamen, 361005 Fujian PR China

**Keywords:** *Actinomycetes*, Menaquinone analysis, Menaquinone identification, Menaquinone extraction

## Abstract

**Background:**

Menaquinones are constituents of prokaryote cell membranes where they play important functions during electron transport. Menaquinone profiles are strongly recommended for species classification when proposing a new *Actinomycetes* taxon. Presently, the most widely used methods to determine menaquinones are based on freeze-dried cells. Taxonomic research in our lab has revealed that menaquinone concentrations are low for some species of the genus *Microbacterium*, leading to difficulties in identifying menaquinones.

**Results:**

Menaquinones extracted using the novel lysozyme-chloroform-methanol (LCM) method were comparable in quality to those obtained using the Collins method, the most widely used method. All tested strains extracted via the LCM method showed higher concentrations of menaquinones than those extracted via the Collins method. For some *Microbacterium* strains, the LCM method exhibited higher sensitivity than the Collins method, and more trace menaquinones were detected with the LCM method than the Collins method. In addition, LCM method is faster than the Collins method because it uses wet cells.

**Conclusion:**

The LCM method is a simple, rapid and efficient technique for the extraction and identification of menaquinones from *Actinomycetes*.

**Supplementary Information:**

The online version contains supplementary material available at 10.1186/s12866-021-02240-z.

## Background

**Fig. 1:**
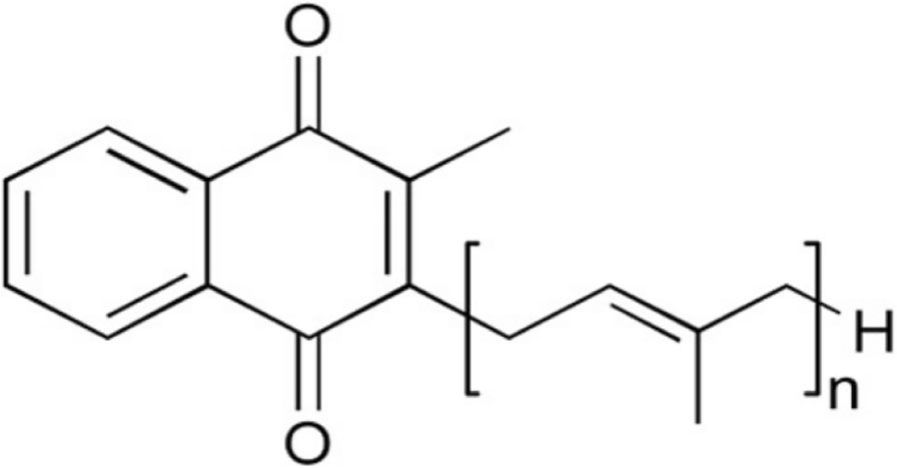
Structure of menaquinones. *n* = 1-15

Menaquinones (MKs), also known as vitamin K_2_, are a large group of fat-soluble compounds composed of a common 2-methyl-1,4-naphthoquinone skeleton with a side chain of different numbers of isoprene units, which are referred to as MK-n (1–15) (Fig. 1) [1]. Since the 1970s, methods for chemotaxonomic characterization of MKs have been used to determine prokaryotic taxonomy [2, 3]. MK extraction and determination methods for *Actinomycetes* were described by Collins et al. in a paper that currently has more than 2000 citations [4–8]. In additional, a respiratory lipoquinone extraction method was reported in 2011 [9], while a screening method for MK-producing strains was reported in 2020 [10]. All of these MK extraction methods use freeze-dried cells. As the most widely used method, we applied the Collins et al. (1977) methodology to test the MKs from *Actinomycetes* strains in our lab. However, some strains within the genus *Microbacterium* show low or non-visible MK bands on a silica gel plate under ultraviolet (UV) radiation. The respiratory quinones from *Microcella putealis* CV-2^T^, CV-40 and AC-30 have also been detected at low concentrations when using 300 mg of freeze-dried cells [11]. Low concentrations of MKs lead to difficulties in identifying MKs. In 2005, a water-soluble MK-7 extraction method for *Bacillus subtilis natto* was reported that demonstrated an increased MK-7 concentration using lysozyme [12]. In 2019, the effect of pretreatment methods for disrupting *Bacillus subtilis natto* cells was reported. Higher yields of MK-7 were obtained with chemical pretreatment methods (including the use of methanol, ethanol, n-propanol and 2-propanol) than enzyme, freezing and heating or ultrasound treatments [13]. In this study, chemical methods using methanol, ethanol and ethyl acetate were initially used to disrupt wet cells of some *Microbacterium* strains before MK extraction. However, non-visible MK bands were found. Fortunately, higher yields of MKs were obtained by disrupting wet cells with lysozyme than those obtained using the Collins et al. (1977) methodology. Here, an improved, simple, rapid (1-day) and high-resolution MK analysis method is reported for *Actinomycetes*.

## Results

### Extraction of menaquinones

The cells used for MK extraction were divided equally into two parts. For the lysozyme-chloroform-methanol (LCM) method proposed in this study, wet cells can be directly used for MK extraction. With this procedure, it takes about 3 h to acquire a crude extract of MKs. However, the Collins method requires hours or even an overnight period to acquire freeze-dried cells and then an overnight extraction is needed to obtain the crude extract of MKs. Therefore, the LCM method is faster than the Collins method.

**Fig. 2:**
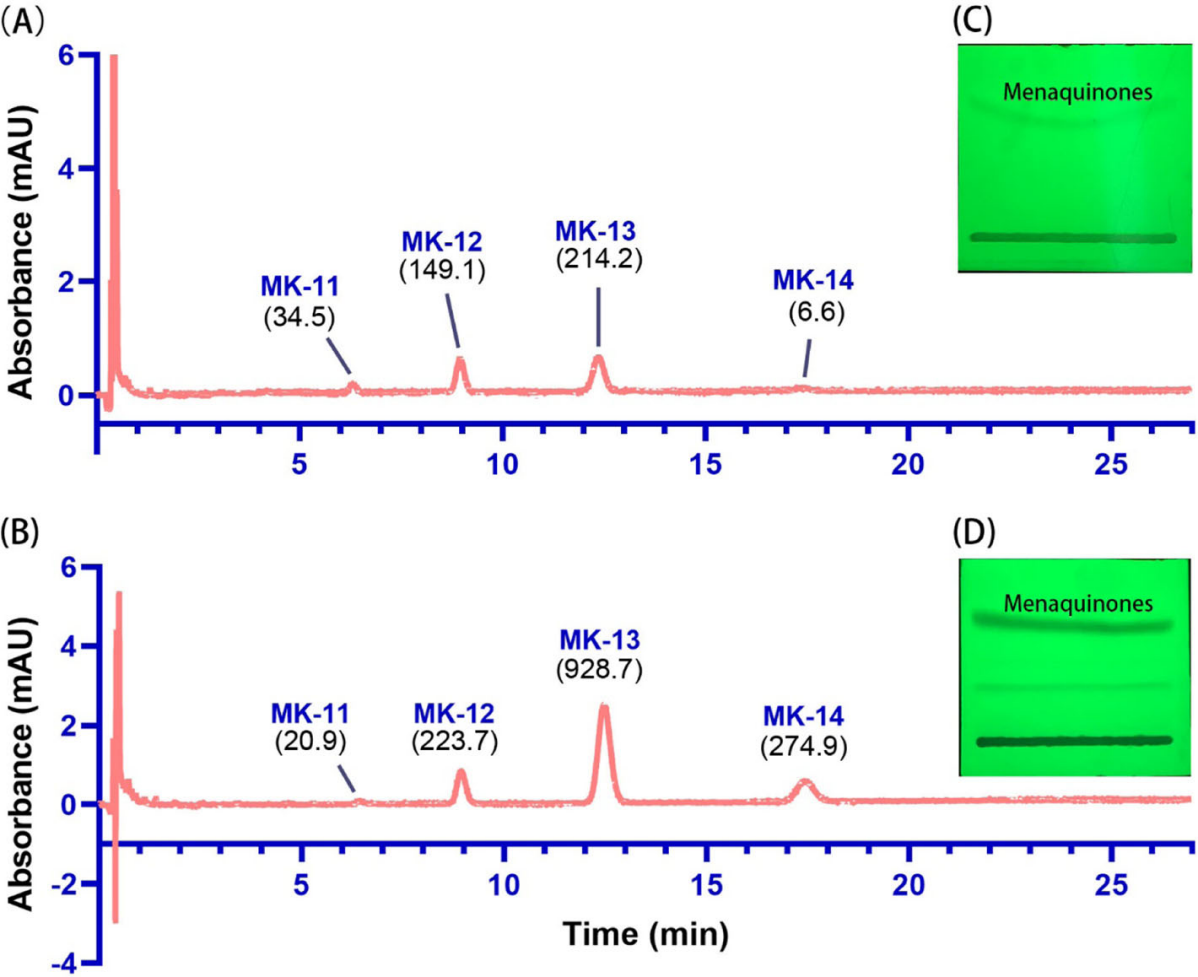
TLC and UPLC-UV analysis of menaquinones (MKs) from *Microbacterium yannicii* JCM 18959^T^ obtained using different MK extraction methods. **A** UPLC-UV analysis of MKs (absorbance at 270 nm) extracted via the Collins method; **B** UPLC-UV analysis of MKs extracted via the LCM method. **C** and **D**: TLC of MKs (exposure to UV radiation at 254 nm) extracted via the Collins method and LCM method, respectively. Strain *M. yannicii* JCM 18959^T^ was chosen as a representative because the fold change of the MK concentration represents the median number of all tested strains. The peak area is shown in parentheses

In addition to the time saving feature, the LCM method obtains higher concentrations of MKs from *Actinomycetes*. In this study, the MK concentrations of the tested strains ranged from 0.063 to 0.921 mg/g dry cell weight (DCW) for the LCM method and 0.001 to 0.591 mg/g DCW for the Collins method (Table 1). When the LCM method was used, the MK concentration from *Saccharopolyspora coralli* E2A^T^ was the lowest (0.063 mg/g DCW) among all the tested strains, but this concentration was higher than that of most strains extracted by the Collins method. *Microbacterium ureisolvens* CFH S00084^T^ showed the lowest MK concentration (0.001 ± 0.001 mg/g DCW) with an almost invisible MK band on thin layer chromatography (TLC) plates using the Collins method. However, when the LCM method was used, the MK concentration of strain CFH S00084^T^ was 355-fold higher (*P* < 0.01) than that of the Collins method. In addition, the MK concentration of *Brachybacterium squillarum* JCM 16464^T^ extracted via the LCM method was 33.2-fold higher (*P* < 0.01) than that of the Collins method. The MK concentrations of other strains extracted by the LCM method were 1.2–17.1-fold higher (*P* < 0.01) than those extracted by the Collins method. As an example, Fig. 2 shows MKs extracted from strain *Microbacterium yannicii* JCM 18959^T^ using both methods and then detected by ultra-performance liquid chromatography (UPLC). Other strains are shown in Supplementary Figures 1 to 11 (Additional file 1). In general, for the same cell biomass, the MK concentration obtained by the LCM method was higher than that obtained by the Collins method.

**Table 1:** Concentration of menaquinones (MKs) extracted by the LCM method and the Collins method

Strains	Menaquinone concentration (mg/g DCW)		Fold change
	LCM method (***n*** = 3)	Collins method (***n*** = 3)	
*Brachybacterium squillarum* JCM 16464^T^	0.664 ± 0.040	0.020 ± 0.003	33.2
*Brevibacterium linens* JCM 1327^T^	0.110 ± 0.011	0.027 ± 0.002	4.1
*Chryseoglobus frigidaquae* JCM 14730^T^	0.151 ± 0.009	0.041 ± 0.007	3.7
*Georgenia subflava* Y32^T^	0.921 ± 0.084	0.054 ± 0.007	17.1
*Janibacter melonis* JCM 16063^T^	0.715 ± 0.079	0.591 ± 0.041	1.2
*Microbacterium yannicii* JCM 18959^T^	0.201 ± 0.014	0.041 ± 0.005	4.9
*Microbacterium ginsengiterrae* JCM 15516^T^	0.322 ± 0.035	0.021 ± 0.002	15.3
*Microbacterium ureisolvens* CFH S00084^T^	0.355 ± 0.022	0.001 ± 0.001	355.0
*Microbacterium hibisci* CCTCC AB 2016180^T^	0.081 ± 0.031	0.046 ± 0.003	1.8
*Nesterenkonia halobia* JCM 15475^T^	0.100 ± 0.010	0.009 ± 0.001	11.1
*Saccharopolyspora coralli* E2A^T^	0.063 ± 0.006	0.012 ± 0.001	5.3
*Yonghaparkia alkaliphile* JCM 15138^T^	0.087 ± 0.001	0.022 ± 0.002	4.0
*Streptomyces indicus* MCCC 1A03308^T^	0.214 ± 0.007	0.163 ± 0.006	1.3

### Identification of menaquinones

**Fig. 3:**
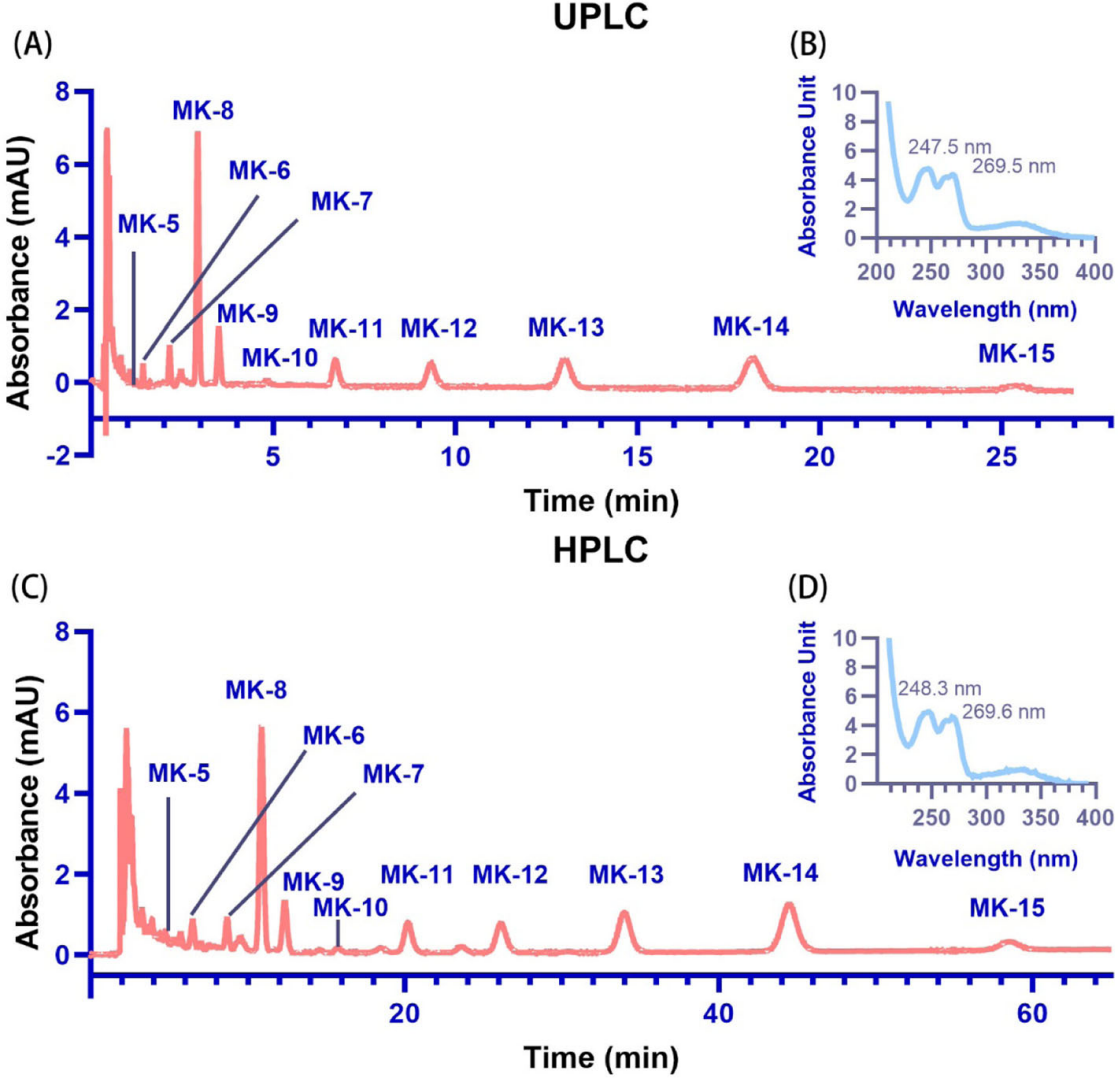
UPLC and HPLC-UV analysis of extracted menaquinones (MK-5 to MK-15). **A** and **C** Representative UPLC and HPLC analyses, respectively, of the MK analogs as measured at an absorbance wavelength of 270 nm. MK-5 to MK-8 were extracted from strain O2; MK-10 to MK-12 were extracted from strain A18JL200; and MK-13 to MK-15 were extracted from strain NY27. MK-9 was used as the MK standard. The absorption spectra of the MK-9 standard and other MKs show the same absorption peaks at 247.5 and 269.5 nm for UPLC (**B**), and 248.3 and 269.6 nm for HPLC (**D**)

**Fig. 4:**
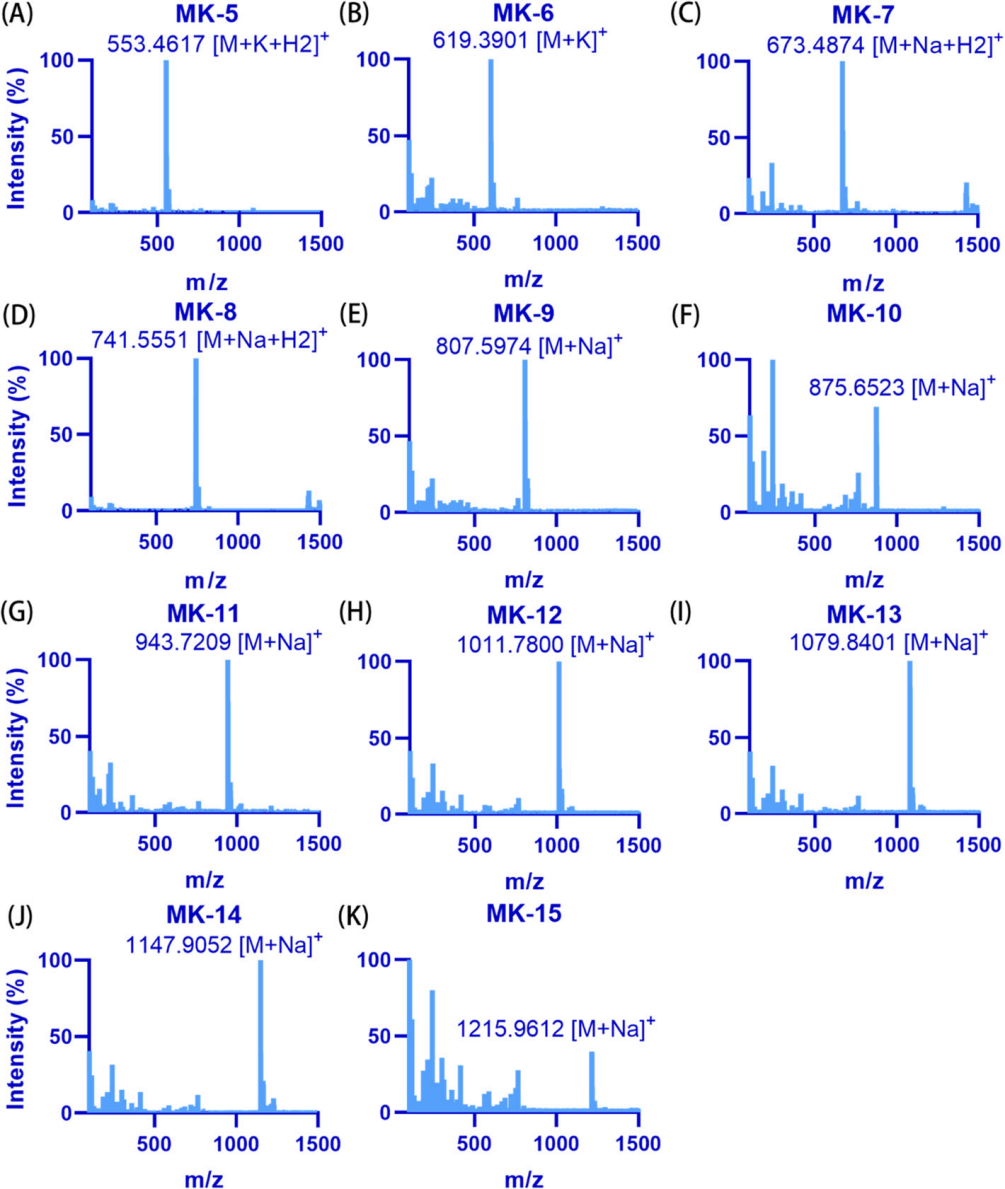
Representative mass spectra of menaquinones (MKs). (A-K) Molecular weight from MK-5 to MK-15. In this system, ion fragments of MK-5 and MK-6 are shown as [M + K + H2]^+^ and [M + K]^+^, respectively; MK-7 and MK-8 are shown as [M + Na + H2]^+^; MK-9 to MK-15 are shown as [M + Na]^+^

**Fig. 5:**
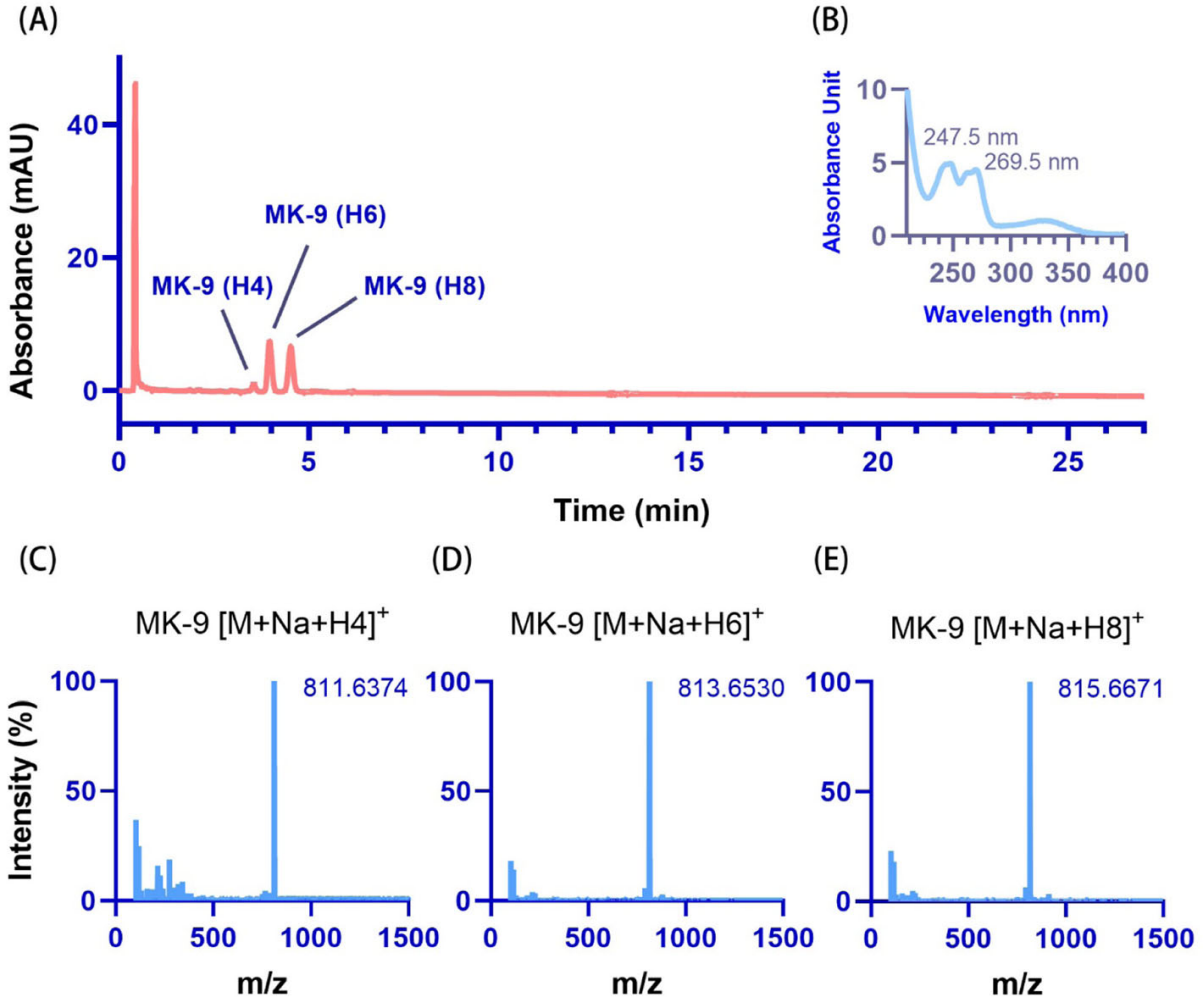
Hydrogenated menaquinones (MKs) from *Streptomyces indicus* MCCC 1A03308^T^ separated using the UPLC-UV/MS system. **A** Representative peaks of MK-9 (H4), MK-9 (H6) and MK-9 (H8) from UPLC. **B** Absorption spectrum of MK-9. **C**-**E** Representative mass spectra of MK-9 (H4), MK-9 (H6) and MK-9 (H8), respectively

As is well known, the UPLC system is faster and more sensitive than the high-performance liquid chromatography (HPLC) system. Thus, the UPLC system was used first for MK identification. The MK-9 standard and MKs extracted from strains O2 (MK-5 to MK-8), A18JL200 (MK-10 to MK-13) and NY27 (MK-13 to MK-15) were mixed as controls for method establishment. MK-5 to MK-15 were observed using the UPLC system at retention times of 1.10, 1.42, 2.16, 2.93, 3.51, 4.84, 6.70, 9.32, 12.98, 18.14 and 25.39 min, respectively (Fig. 3A). Because UPLC equipment may not be available in every laboratory, HPLC was also used for MK separation in this study. MK-5 to MK-15 were separated by the HPLC system with retention times of 5.27, 6.49, 8.68, 10.88, 12.36, 15.71, 20.20, 26.12, 33.98, 44.46 and 58.48 min, respectively (Fig. 3C). In this study, both the HPLC and UPLC systems separated MK-5 to MK-15 well, with no significant difference between the two systems in their ability to separate these MKs. A larger sample volume (10 μL) may be used to acquire a better signal for the HPLC system. The absorption spectrum of each MK exhibited two peaks: 247.5 and 269.5 nm for the UPLC system (Fig. 3B) and 248.3 and 269.6 nm for the HPLC system (Fig. 3D). The molecular mass of the MKs detected by mass spectrometer (MS), as shown by the mass spectra (Fig. 4), most accurately matched the ion fragment of [M + Na]^+^ (Table 2). According to the spectral absorption peak and molecular mass, we could confirm each of the MKs. Unsaturated MKs were observed for most of the strains in this study. Hydrogenated MKs of MK-5 (H2), MK-7 (H2) and MK-8 (H2) were observed in strain O2; MK-9 (H2, H4) was observed in strain E2A^T^; and MK-9 (H4, H6, H8) was observed in strain MCCC 1A03308^T^. MK-9 (H4), MK-9 (H6) and MK-9 (H8) from *Streptomyces indicus* MCCC 1A03308^T^ extracted via the LCM method separated well using the UPLC system, showing retention times of 3.54, 3.97 and 4.52 min, respectively (Fig. 5). Hydrogenated MKs also separated well using the HPLC system.

**Table 2:** Reference molecular formulas and molecular weights for each menaquinone (MK) ion fragment

MK	Molecular formula	[M]^**+**^	[M + H]^**+**^	[M + Na]^**+**^	[M + K]^**+**^
MK-1	C_16_H_16_O_2_	240.115	241.1229	263.1048	279.0787
MK-2	C_21_H_24_O_2_	308.1776	309.1855	331.1674	347.1413
MK-3	C_26_H_32_O_2_	376.2402	377.2481	399.2300	415.2039
MK-4	C_31_H_40_O_2_	444.3028	445.3107	467.2926	483.2665
MK-5	C_36_H_48_O_2_	512.3654	513.3733	535.3552	551.3291
MK-6	C_41_H_56_O_2_	580.4280	581.4359	603.4178	619.3917
MK-7	C_46_H_64_O_2_	648.4906	649.4985	671.4804	687.4543
MK-8	C_51_H_72_O_2_	716.5532	717.5611	739.5430	755.5169
MK-9	C_56_H_80_O_2_	784.6158	785.6237	807.6056	823.5795
MK-10	C_61_H_88_O_2_	852.6784	853.6863	875.6682	891.6421
MK-11	C_66_H_96_O_2_	920.7410	921.7489	943.7308	959.7047
MK-12	C_71_H_104_O_2_	988.8036	989.8115	1011.7934	1027.7673
MK-13	C_76_H_112_O_2_	1056.8662	1057.8741	1079.856	1095.8299
MK-14	C_81_H_120_O_2_	1124.9288	1125.9367	1147.9186	1163.8925
MK-15	C_86_H_128_O_2_	1192.9914	1193.9993	1215.9812	1231.9551

MKs of the tested strains using the LCM and Collins methods are shown in Table 3. The corresponding UPLC-UV data for these strains are shown in Supplementary Figures1 to 11 (Additional file 1). The main MKs of all the strains detected using both methods were primarily the same as reported (Table 3). The MK composition of the tested strains extracted with both the LCM and Collins methods were the same except for *M. ureisolvens* CFH S00084^T^ and *M. ginsengiterrae* JCM 15516^T^. Specifically, MK-12 and MK-13 were the observed MKs from strains CFH S000084^T^ and JCM 15516^T^ using the Collins method; however, along with MK-12 and MK-13, MK-11 and MK-14 were also detected with significant peaks in these two strains when using the LCM method (Additional file 1: Supplementary Figure 6 and 7).

**Table 3:** Menaquinone (MK) components from type strains as extracted by the lysozyme-chloroform-methanol (LCM) method and the Collins method, as well as those previously reported in the literature. Low-concentration MKs (< 10%) are shown in parentheses

Strains	LCM method	Collins method	Reported menaquinones
*Brachybacterium squillarum* JCM 16464^T^	(MK-6), MK-7, (MK-8)	(MK-6), MK-7, (MK-8)	MK-7 [14]
*Brevibacterium linens* JCM 1327^T^	(MK-7), MK-8, (MK-9)	(MK-7), MK-8, (MK-9)	MK-8 [15]
*Chryseoglobus frigidaquae* JCM 14730^T^	(MK-11), MK-12, MK-13, MK-14	(MK-11), MK-12, MK-13, MK-14	MK-12, MK-13, MK-14 [16]
*Georgenia subflava* Y32^T^	(MK-6), MK-7, (MK-8)	(MK-6), MK-7, (MK-8)	MK-7, MK-8 [17]
*Janibacter melonis* JCM 16063^T^	(MK-7), MK-8	(MK-7), MK-8	MK-8 [18]
*Microbacterium yannicii* JCM 18959^T^	(MK-11), MK-12, MK-13, MK-14	(MK-11), MK-12, MK-13, (MK-14)	MK-11, MK-12, MK-13 [19]
*Microbacterium ginsengiterrae* JCM 15516^T^	(MK-11), MK-12, MK-13, (MK-14)	MK-12, MK-13	MK-12, MK-13 [20]
*Microbacterium ureisolvens* CFH S00084^T^	(MK-11), MK-12, MK-13, (MK-14)	MK-12, MK-13	MK-11, MK-12, MK-13 [21]
*Microbacterium hibisci* CCTCC AB 2016180^T^	(MK-11), MK-12, MK-13, (MK-14)	(MK11), MK-12, MK-13, (MK-14)	MK-12, MK-13 [22]
*Nesterenkonia halobia* JCM 15475^T^	MK-7, MK-8, MK-9	MK-7, MK-8, MK-9	MK-8, MK-9 [23]
*Saccharopolyspora coralli* E2A^T^	MK-9 (H2, H4)	MK-9 (H2, H4)	MK-9 [24]
*Yonghaparkia alkaliphile* JCM 15138^T^	MK-11, MK-12, MK-13	MK-11, MK-12, MK-13	MK-12 [25]
*Streptomyces indicus* MCCC 1A03308^T^	MK-9 (H4, H6, H8)	MK-9 (H4, H6, H8)	MK-9 (H4, H6, H8) [26]

## Discussion

To validate the LCM method, the popular MK extraction protocol described by Collins et al. (1977) was used as a control to test the reliability of our MK extraction method from wet cells. During exploratory development of the LCM method, chloroform:methanol (2:1 v/v) was first used to extract MKs directly from wet cells without a lysozyme pretreatment; however, no MKs were obtained from *Microbacterium* strains. Besides wet cells, freeze-dried cells can also be used for the LCM method, and there is no significant difference in MK concentrations between the wet cells and freeze-dried cells. However, the use of freeze-dried cells will extend the experimental time. For the lysozyme treatment conditions, the optimal concentration of lysozyme and incubation time are also different for different strains. Generally, most of the cells can be lysed with a final lysozyme concentration of 1 mg/mL and an incubation time of 30 min to 1 h [27]. If the concentration of MKs is low for LCM method, increasing the concentration of lysozyme and prolonging the treatment time will provide better results. Water in the lysozyme-treated cells should be removed using methanol or ethanol before the chloroform-methanol extraction step, otherwise the extraction efficiency will be reduced. TLC is an important purification and verification step during the MK extraction. Purified MKs were easily identified. However, if the MK concentration is low, the MK band (Rf ≈ 0.7) on the TLC plate will be weak and not easily observed. As a result, minor MK components would not be detected by UPLC/MS or HPLC/MS. Accordingly, the MK intensity can be increased by concentrating the extract or increasing the injection volume. Better separation of MKs can be obtained using the ACQUITY UPLC® HSS C18 column (1.8 μm 2.1 × 100 mm) with the UPLC system. The elution time is important for MK analysis. In order to avoid invalid elution, it is better to use an MK-15 standard as a reference for the analysis of UPLC or HPLC results, especially when the strain contains MK-12 to MK-15. In addition, MKs are delicate components that are easily degraded. To prevent photo-oxidation, strong light should be avoided during the MK extraction process.

Overall, the results obtained using the LCM method were comparable in quality to those obtained using the standard freeze-dried approach. All tested strains extracted via the LCM method showed higher concentrations of MKs when compared with those extracted by the Collins method. Due to the high efficiency of MK extraction, the LCM method may serve as a general method for MK identification and screening of vitamin K_2_-producing *Actinomycetes* strains.

## Conclusion

A simple, rapid and efficient method for identifying MKs from *Actinomycetes* in wet biomass was established. Compared with the Collins method, currently the most widely used method, MK extraction via the LCM method is more sensitive and time-saving.

## Methods

### Chemicals and reagents

All reagents and solvents used during the extraction were analytical grade. Strains were incubated in 2216E medium (pH 7.4 ~ 7.6), which was composed of 5.0 g/L peptone, 1.0 g/L yeast extract, 0.1 g/L FeC_6_H_5_O_7_, 19.45 g/L NaCl, 5.98 g/L MgCl_2_, 3.24 g/L Na_2_SO_4_, 1.8 g/L CaCl_2_, 0.55 g/L KCl, 0.16 g/L Na_2_CO_3_, 0.08 g/L KBr, 0.034 g/L SrCl_2_, 0.022 g/L H_3_BO_3_, 0.004 g/L Na_2_SiO_3_, 0.0024 g/L NaF, 0.0016 g/L NH_4_NO_3_ and 0.008 g/L Na_2_HPO_3_. Lysozyme (Solarbio Science & Technology, Beijing, China) was used for cell wall digestion. Tris-HCl buffer (10 mM, pH 7.4), chloroform-methanol (2:1 v/v) and hexane-diethyl ether (85:15, v/v) were used for MK extraction. These organic reagents were purchased from Xilong Scientific Co., Ltd., Guangzhou, China. The MK-9 standard was purchased from GLPBIO, Montclair, CA, USA. Mass spectrometry-grade methanol (Sigma-Aldrich, St. Louis, MO, USA) and isopropanol (Fisher Chemical, Thermo-Fisher Scientific Inc.) were used for high-performance liquid chromatography (HPLC), ultra-performance liquid chromatography (UPLC), and mass spectrometer (MS).

### Strains for menaquinone analysis

*Microbacterium yannicii* JCM 18959^T^, *Microbacterium ginsengiterrae* JCM 15516^T^, *Brachybacterium squillarum* JCM 16464^T^, *Nesterenkonia halobia* JCM 15475^T^, *Chryseoglobus frigidaquae* JCM 14730^T^, *Brevibacterium linens* JCM 1327^T^, *Yonghaparkia alkaliphila* JCM 15138^T^ and *Janibacter melonis* JCM 16063^T^ were purchased from the Japan Collection of Microorganisms (JCM). *Streptomyces indicus* MCCC 1A03308^T^ was purchased from the Marine Culture Collection of China (MCCC). *Microbacterium hibisci* CCTCC AB 2016180^T^ was purchased from the China Center for Type Culture Collection (CCTCC). *Microbacterium. ureisolvens* CFH S00084^T^ was provided by Guo-Xing Nie, College of Fisheries, Henan Normal University, China. *Saccharopolyspora coralli* E2A^T^, *Georgenia subflava* Y32^T^, *Microbacterium* sp. NY27, *Microbacterium* sp. A18JL200 and *Brevibacterium* sp. O2 were isolated by our lab.

### Equipment

Test tubes and 1-L Erlenmeyer flasks were used for seed cultures and strain fermentation. Cell collection and MK extraction were performed with 50-mL centrifuge tubes (Sangon Biotech Co., Ltd., Shanghai, China) and an Eppendorf 5804R centrifuge (Eppendorf China Co., Ltd.). Lysozyme digestion was done in an electro-thermostatic water bath (YiHeng technical Co., Ltd., Shanghai, China). Extracted MKs were dried using a rotary evaporator (Heidolph Instruments GmbH & CO. KG). Thin-layer chromatography (TLC) was performed with 0.4–0.5 mm layers of silica-gel HF_254_ (10 × 10 cm) and a glass developing tank (Jiangyou Silica gel Development Co., Ltd., Yantai, China). MKs were observed under UV radiation with an UV analyzer (Chi Tang Industrial Co., Ltd., China). Organic solvent was filtered with 2-mL syringes (Kangyou Medical Equipment Co., Ltd., Jiangsu, China) and 0.22 μm/13 mm nylon syringe filters (Sangon Biotech Co., Ltd., Shanghai, China). Samples were injected into the HPLC system (Waters Alliance e2695) and the Waters ACQUITY UPLC® system from 2-mL amber screw top autosampler vials (Thermo-Fisher Scientific Inc.). The HPLC system was equipped with a 2998 PDA detector and a SunFire™ C18 column (5 μm 4.6 × 150 mm). The UPLC system was equipped with a C18 reversed-phase column (1.7 μm 2.1 × 50 mm, ACQUITY UPLC® BEH C18), a PDA eλ detector and a high-resolution MS (Xevo G2 Q-TOF with electrospray ionization (ESI)).

### Strain cultivation and collection

All strains used in this research were cultured on 2216E agar plates. A single colony was incubated in test tube containing 5 mL of 2216E medium at 28 °C with 150 rpm shaking until an optical density (OD at 600 nm) of 0.6 was reached. Next, 2 mL of seed culture was added into 200 mL of 2216E medium in 1-L Erlenmeyer flasks and incubated for 3 to 4 days on a rotary shaker at 150 rpm and 28 °C. Cells were divided into two equal parts for different MK extraction methods and then collected in 50-mL centrifugal tubes using an Eppendorf 5804R centrifuge at 6000×g for 15 min. Three independent replicates of strain cultivation and collection were carried out.

### Lysozyme-chloroform-methanol extraction method

Since the thick cell wall of *Actinomycetes* can resist organic solvent extraction, an improved method named the lysozyme-chloroform-methanol (LCM) method was used to extract MKs. For this method, lysozyme was used to break the cell walls, followed by a chloroform-methanol (2:1 v/v) extraction. After concentration using a rotary evaporator and purification via thin-layer chromatography (TLC), MKs were observed using a UV analyzer at 254 nm and eluted using isopropanol. Three replicates were carried out for the LCM method in this study.

Extraction steps were as follows:

1. Wet cellular mass (0.7–1.0 g) was acquired for each strain as described above.
2. Cells were washed twice with 20 mL of 10 mM Tris-HCl buffer (pH 7.4) to avoid media contamination, and then they were suspended in 50 mL of 10 mM Tris-HCl buffer (pH 7.4) with 50 mg of lysozyme. The solution was mixed well by shanking for 1 min and incubated in a 37 °C water bath for 1 h (with 1 min of shaking every 10 min) to digest the cell wall. Next, the mixture was centrifuged for 15 min at 6000×g to collect the lysozyme-treated cells. (Note: the cell walls of some *Actinomycetes* are easy digested by lysozyme, forming a colloidal solution that is hard to centrifuge; thus, the next water removal step is important.)
3. Lysozyme-treated cells were washed with 5 mL of methanol (or ethanol) to remove water. The methanol (or ethanol) should be collected because it can dissolve some MKs. (Note: for easily-digested *Actinomycetes*, remove the water as much as possible after centrifugation and then add an equal volume of methanol (or ethanol) to the remaining solution. Shake several times and then centrifuge for 15 min at 6000×g. Cells can then be collected and washed with 5 mL of methanol (or ethanol). The top water-methanol solution should also be collected.)
4. Chloroform/methanol (10 mL, 2:1 v/v) was added to the cells and then shaken for 1 min to extract MKs. The chloroform-methanol extraction was repeated three times. Approximately 30 mL of crude extract was acquired.
5. The methanol (or ethanol) and chloroform-methanol extracts were collected and dried using a rotary evaporator at 35 °C. The dry product was then dissolved with 500 μL of chloroform-methanol (2:1 v/v) three to four times.
6. The chloroform-methanol-dissolved crude extract was purified by TLC, which was performed using 0.4–0.5 mm layers of silica gel HF_254_ (10 × 10 cm) and a developing solvent consisting of hexane/diethyl ether (85:15, v/v). MKs are routinely detected via TLC using brief irradiation with short-wave UV light (254 nm). In this system, the MKs migrate about Rf ≈ 0.7. The MK band was collected and eluted using 1.5 mL isopropanol.
7. Isopropanol-eluted MKs were filtered with 0.22 μm nylon syringe filters into 2-mL amber screw top autosampler vials and then examined by UPLC-UV/MS or HPLC-UV/MS

### Collins et al. (1977) method for menaquinone extraction

Tubes containing wet cells were freeze-dried for 12 h. Dried cells were mixed with 20 mL of chloroform/methanol (2:1, v/v). The suspension was then continuously stirred overnight. The cells were then removed by filtration and the extract was dried by evaporation under reduced pressure at a low temperature (35 °C). Analytical TLC of quinones was performed using 0.4–0.5 mm layers of silica gel HF_254_ and a developing solvent consisting of hexane/diethyl ether (85:15, v/v). Isopropanol was used to elute the quinones from the silica gel. Isopropanol-eluted MKs were filtered with 0.22 μm nylon syringe filters into 2-mL amber screw top autosampler vials and then examined by UPLC-UV/MS or HPLC-UV/MS. Three replicates were carried out for the Collins method.

### Identification of menaquinones using the HPLC and UPLC-UV/MS systems

For HPLC analysis, the mobile phase was a methanol/isopropanol (1:1, v/v) solution; the column temperature was set to 35 °C; the flow rate was 0.75 mL/min for 65 min; and the injection volume was 10 μL. For UV analysis, the wavelength was set as 270 nm and the 3D spectrum range was from 210 to 400 nm. For UPLC analysis, the mobile phase was methanol/isopropanol (3:1, v/v); the column temperature was set to 35 °C; the flow rate was 0.3 mL/min for 27 min; and the injection volume was 1 μL. For UV analysis, the wavelength was set as 270 nm and the 3D spectrum range was from 210 to 400 nm. For MS analysis, electrospray ionization (ESI) was conducted in positive ion mode. A scan range of 100 to 1500 m/z was used. The molecular weights of the MKs were then calculated using the m/z ratios of their ion fragments (e.g., [M]^+^, [M + H]^+^ and/or [M + Na]^+^), and their accurate molecular formulae and chemical structures were subsequently confirmed. MK-9 was used as a reference.

### Quantification of menaquinones

Three replicates were carried out to compare the LCM method and the Collins method. For each replicate, cells of each strain were divided into two equal parts, one for the LCM method and the other for the Collins method. Tubes containing wet cells for the Collins method were freeze-dried for 12 h. The dry cell weight (DCW) was calculated as: DCW = the total weight of the centrifuge tube containing dry cells - weight of the empty centrifuge tube. The DCW used in the LCM method was based on that used for the Collins method. MK concentrations were measured via the UPLC system, using MK-9 as the standard, as previously described [28]. The yield was expressed as mg menaquinones/g dry biomass. The data were analyzed for comparison using SPSS 19 software and values are reported as mean ± standard deviation (SD) (*n* = 3). One-way analysis of variance (ANOVA) was used to test the significance of the two methods.

## Supplementary Information

**Additional file 1.**

## Data Availability

All data generated or analyzed during this study are included in this published article [and its supplementary information files].

## Abbreviations

MK: Menaquinone
LCM: Lysozyme-chloroform-methanol;
UV: Ultraviolet
DCW: Dry cell weight
TLC: Thin layer chromatography
UPLC: Ultra-performance liquid chromatography
HPLC: High-performance liquid chromatography
MS: Mass spectrometer
JCM: Japan Collection of Microorganisms
MCCC: Marine Culture Collection of China
CCTCC: China Center for Type Culture Collection
SD: Standard deviation
ANOVA: One-way analysis of variance.
