# Supplementary material for: A rapid and efficient method for the extraction and identification of menaquinones from Actinomycetes in wet biomass
Source: BMC Microbiol. 2021 Jun 9;21:175. doi: 10.1186/s12866-021-02240-z (PMC8188722; doi:10.1186/s12866-021-02240-z)
Supplement: Supplementary file 1 — Additional file 1. [file 12866_2021_2240_MOESM1_ESM.docx]

***Supplementary Material***

- **UPLC-UV of menaquinones extracted from type strains with the Collins method and Lysozyme-Chloroform-Methanol (LCM) method**

Menaquinones of type strains *Brachybacterium squillarum* JCM 16464^T^, *Brevibacterium linens*JCM 1327^T^, *Chryseoglobus frigidaquae* JCM 14730^T^, *Georgenia subflava* Y32^T^, *Janibacter melonis* JCM 16063^T^, *Microbacterium ginsengiterrae* JCM 15516^T^, *Microbacterium ureisolvens* CFH S00084^T^, *Microbacterium hibisci* CCTCC AB 2016180^T^, *Nesterenkonia halobia*JCM 15475^T^, *Saccharopolyspora coralli* E2A^T^ and *Yonghaparkia alkaliphila* JCM 15138^T^ extracted with the Collins method and LCM method were measured by UPLC-UV/MS system. MKs of each peak was identified by 3D absorption spectrum and mass spectrum. MK composition was shown in Supplementary Figure 1 to 11.

- **Supplementary Figures**


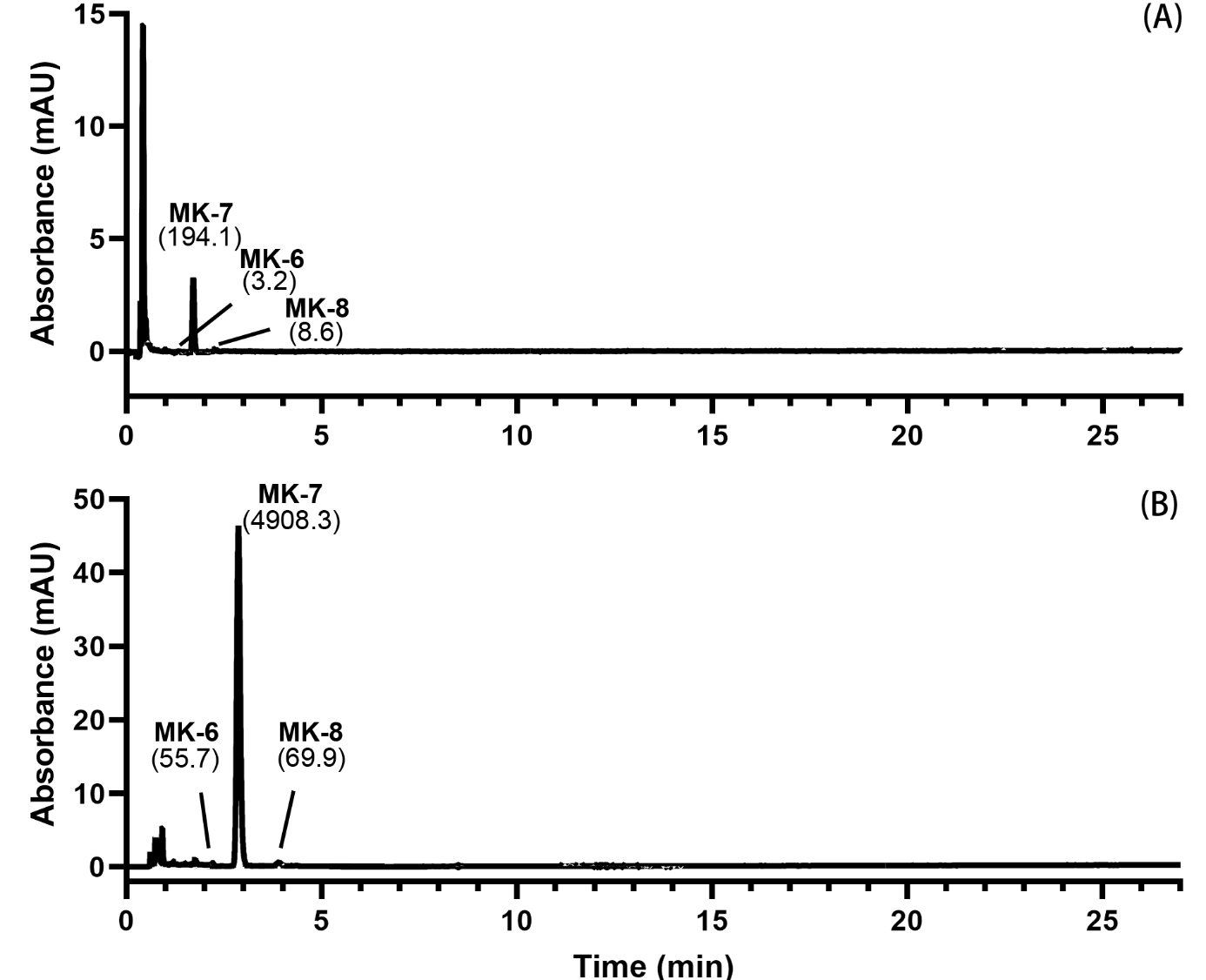


**Supplementary Figure 1.** UPLC-UV analysis of menaquinones (absorbance at 270 nm) extracted from type strain JCM 16464^T^. (A) and (B) Representative UPLC-UV analysis of menaquinones extracted with the Collins method and LCM method, respectively. The peak area is shown in parentheses.


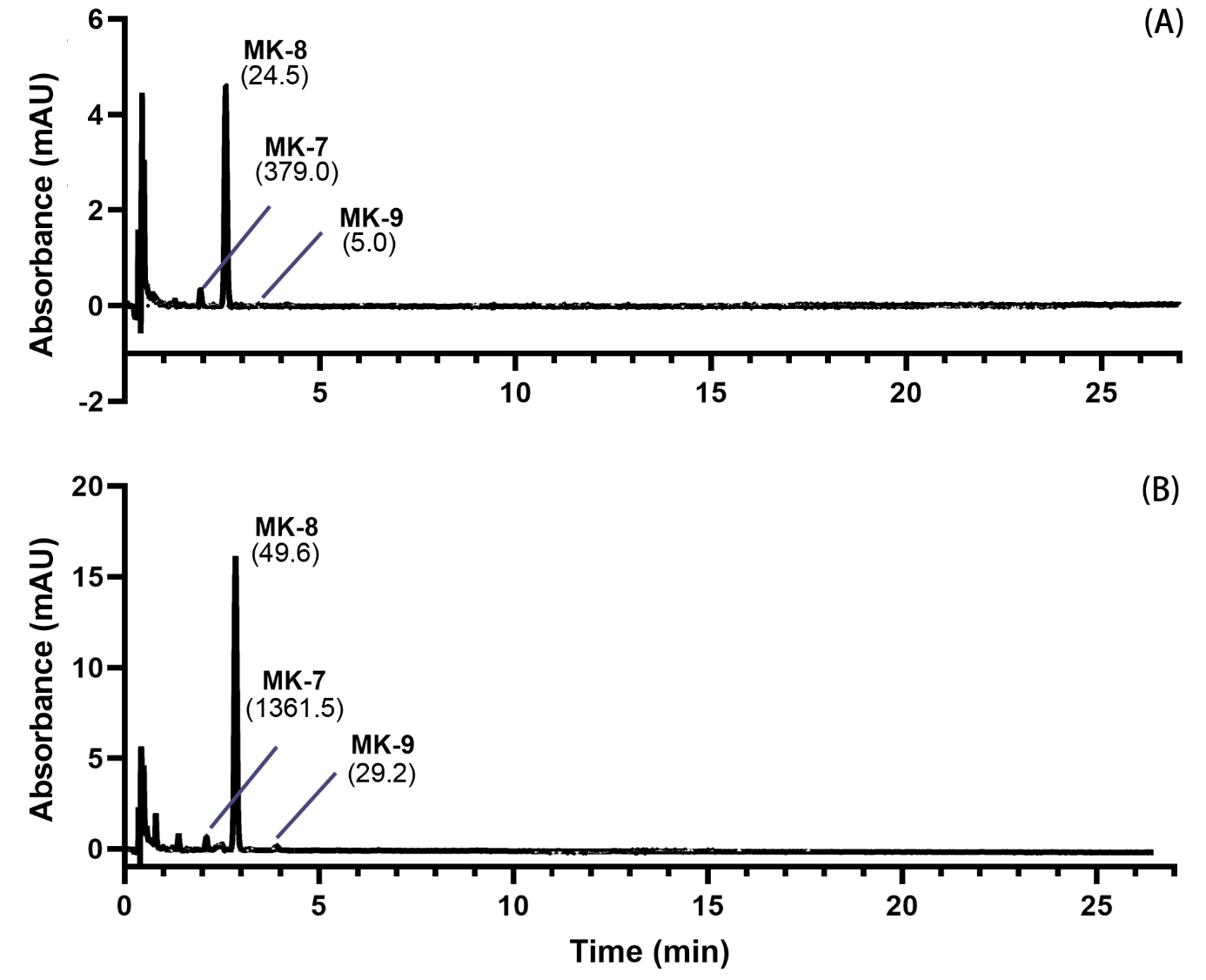
**Supplementary Figure 2.** UPLC-UV analysis of menaquinones (absorbance at 270 nm) extracted from type strain JCM 1327^T^. (A) and (B) Representative UPLC-UV analysis of menaquinones extracted with the Collins method and LCM method, respectively. The peak area is shown in parentheses.


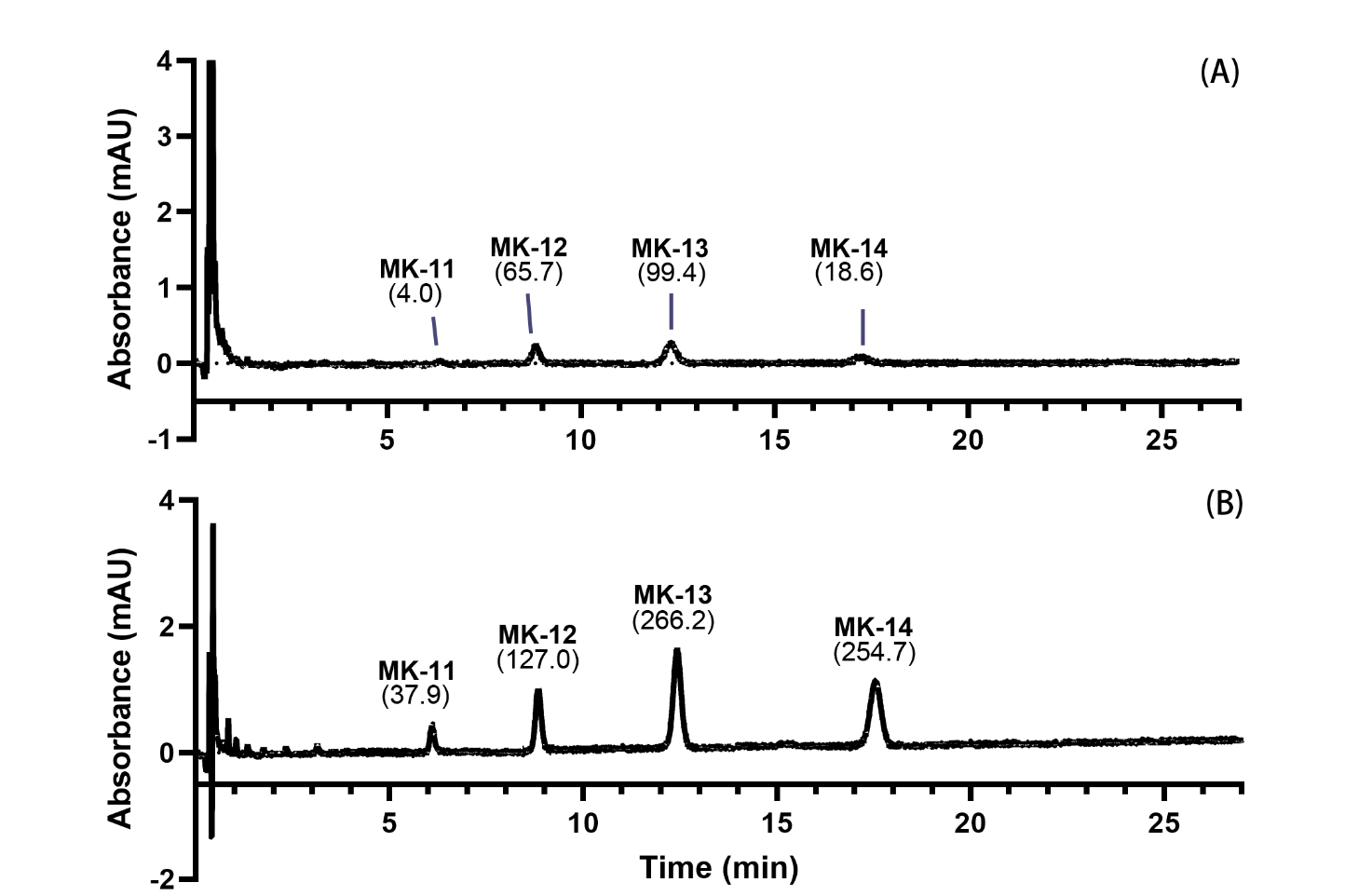
**Supplementary Figure 3.** UPLC-UV analysis of menaquinones (absorbance at 270 nm) extracted from type strain JCM 14730^T^. (A) and (B) Representative UPLC-UV analysis of menaquinones extracted with the Collins method and LCM method, respectively. The peak area is shown in parentheses.


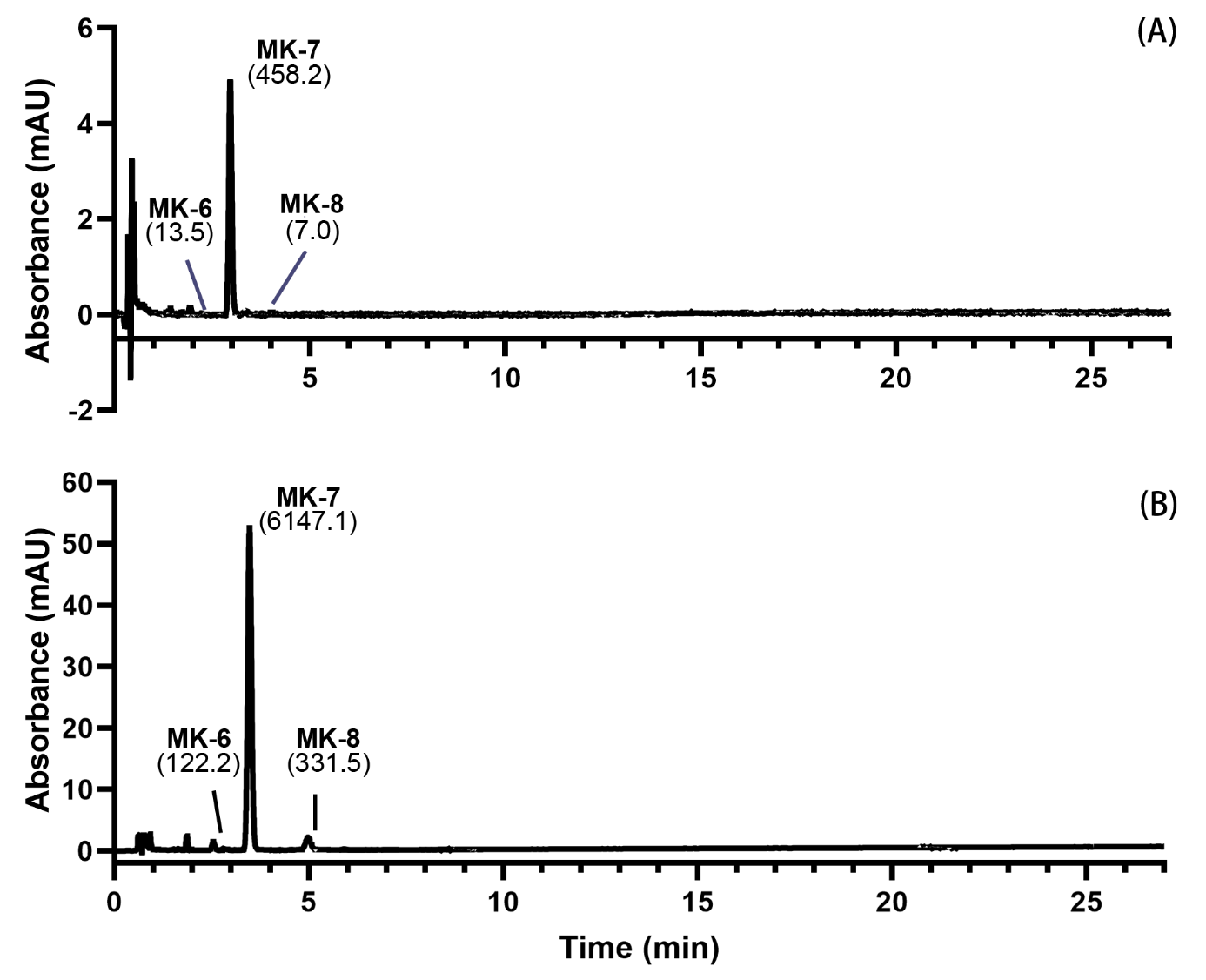
**Supplementary Figure 4.** UPLC-UV analysis of menaquinones (absorbance at 270 nm) extracted from type strain Y32^T^. (A) and (B) Representative UPLC-UV analysis of menaquinones extracted with the Collins method and LCM method, respectively. The peak area is shown in parentheses.


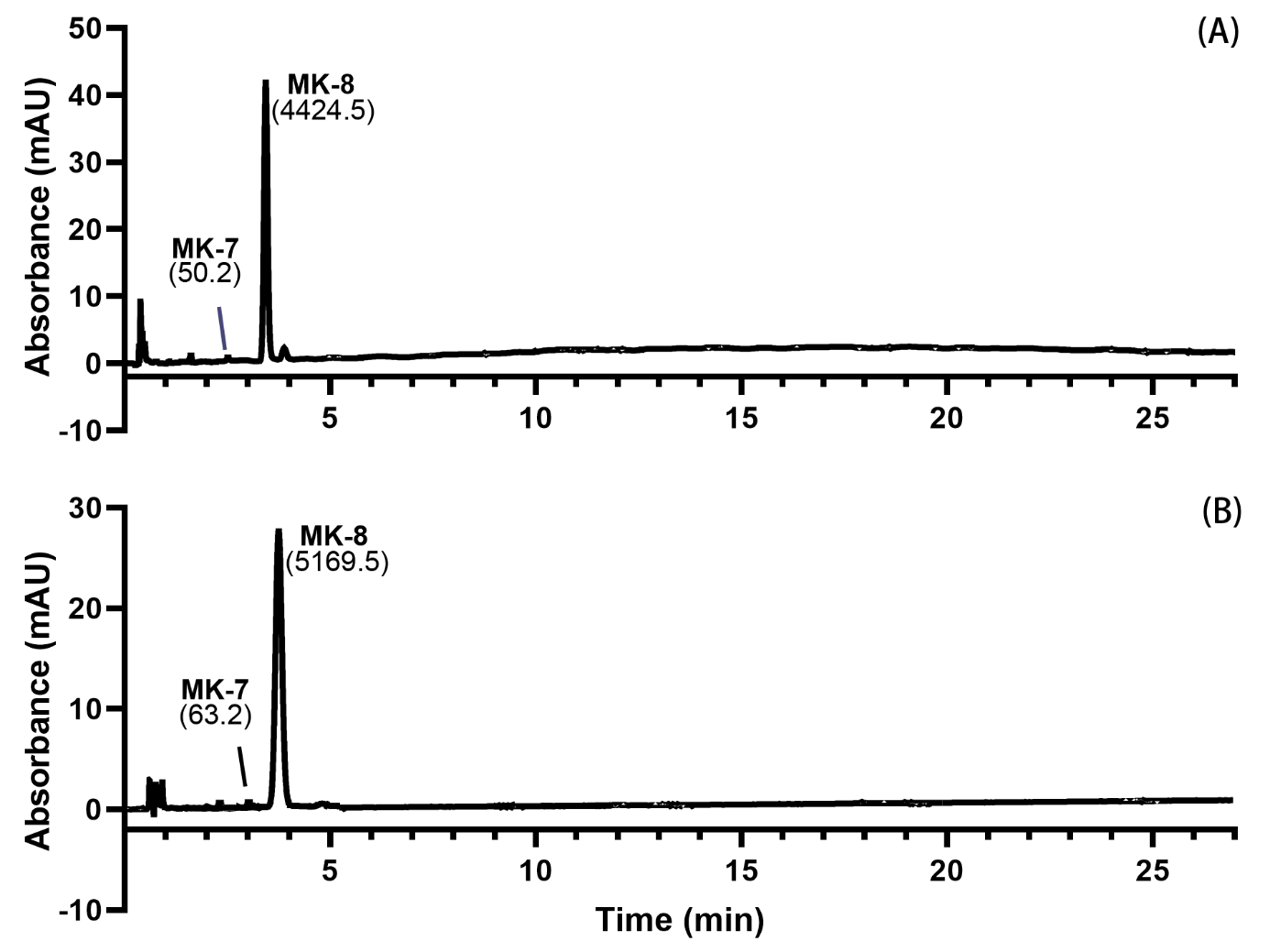
**Supplementary Figure 5.** UPLC-UV analysis of menaquinones (absorbance at 270 nm) extracted from type strain JCM 16063^T^. (A) and (B) Representative UPLC-UV analysis of menaquinones extracted with the Collins method and LCM method, respectively. The peak area is shown in parentheses.


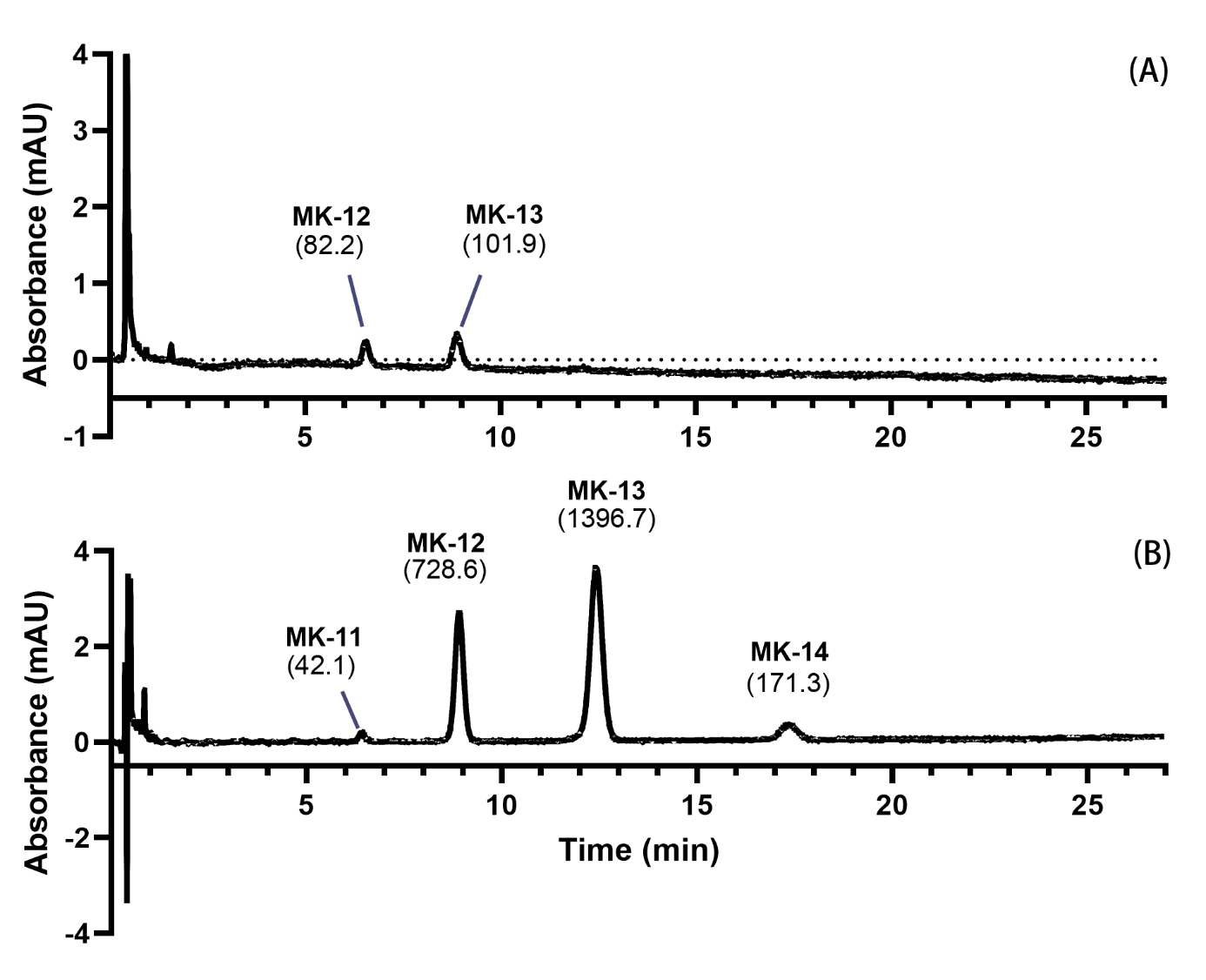
**Supplementary Figure 6.** UPLC-UV analysis of menaquinones (absorbance at 270 nm) extracted from type strain JCM 15516^T^. (A) and (B) Representative UPLC-UV analysis of menaquinones extracted with the Collins method and LCM method, respectively. The peak area is shown in parentheses.


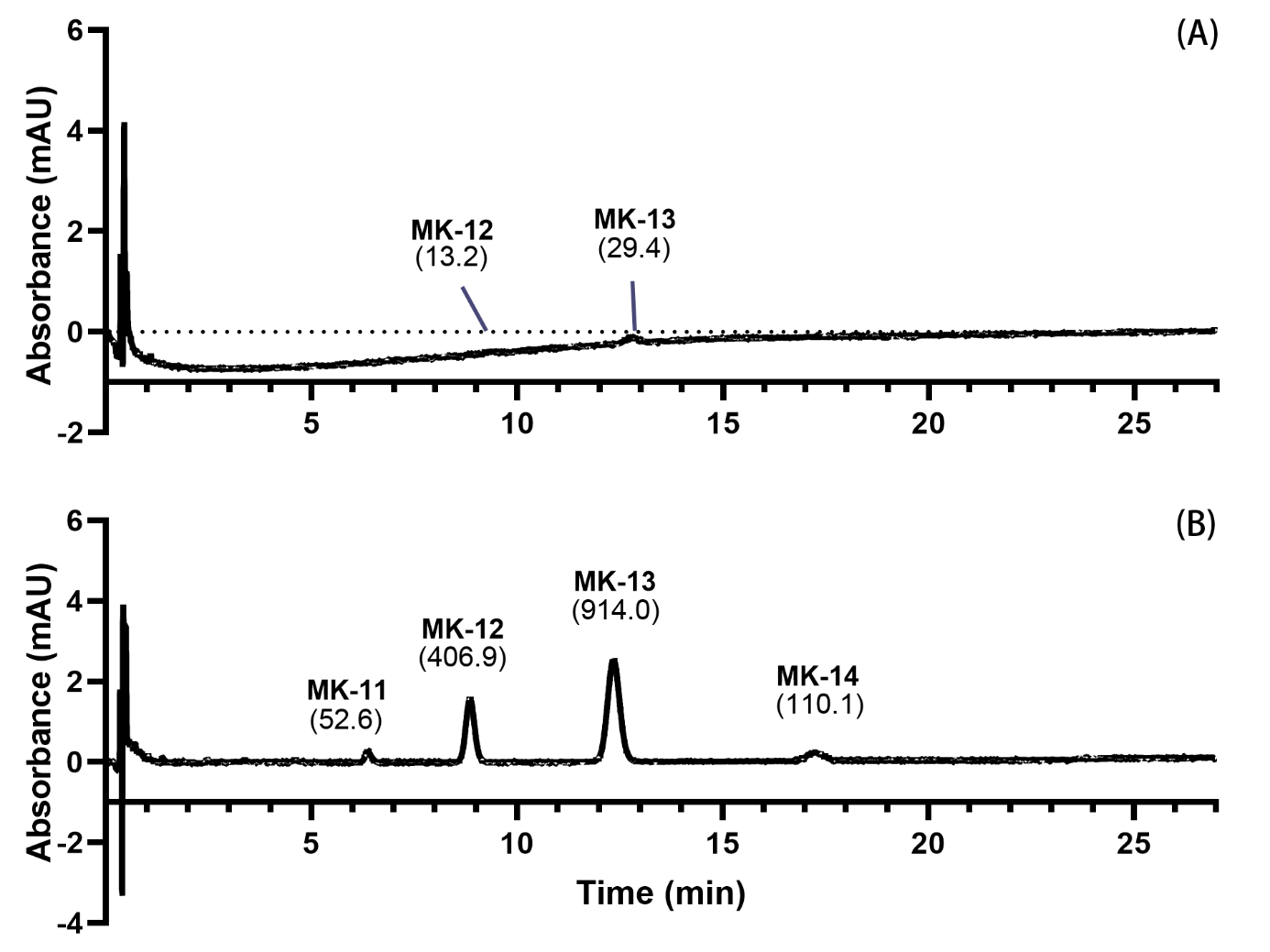
**Supplementary Figure 7.** UPLC-UV analysis of menaquinones (absorbance at 270 nm) extracted from type strain CFH S00084^T^. (A) and (B) Representative UPLC-UV analysis of menaquinones extracted with the Collins method and LCM method, respectively. The peak area is shown in parentheses.


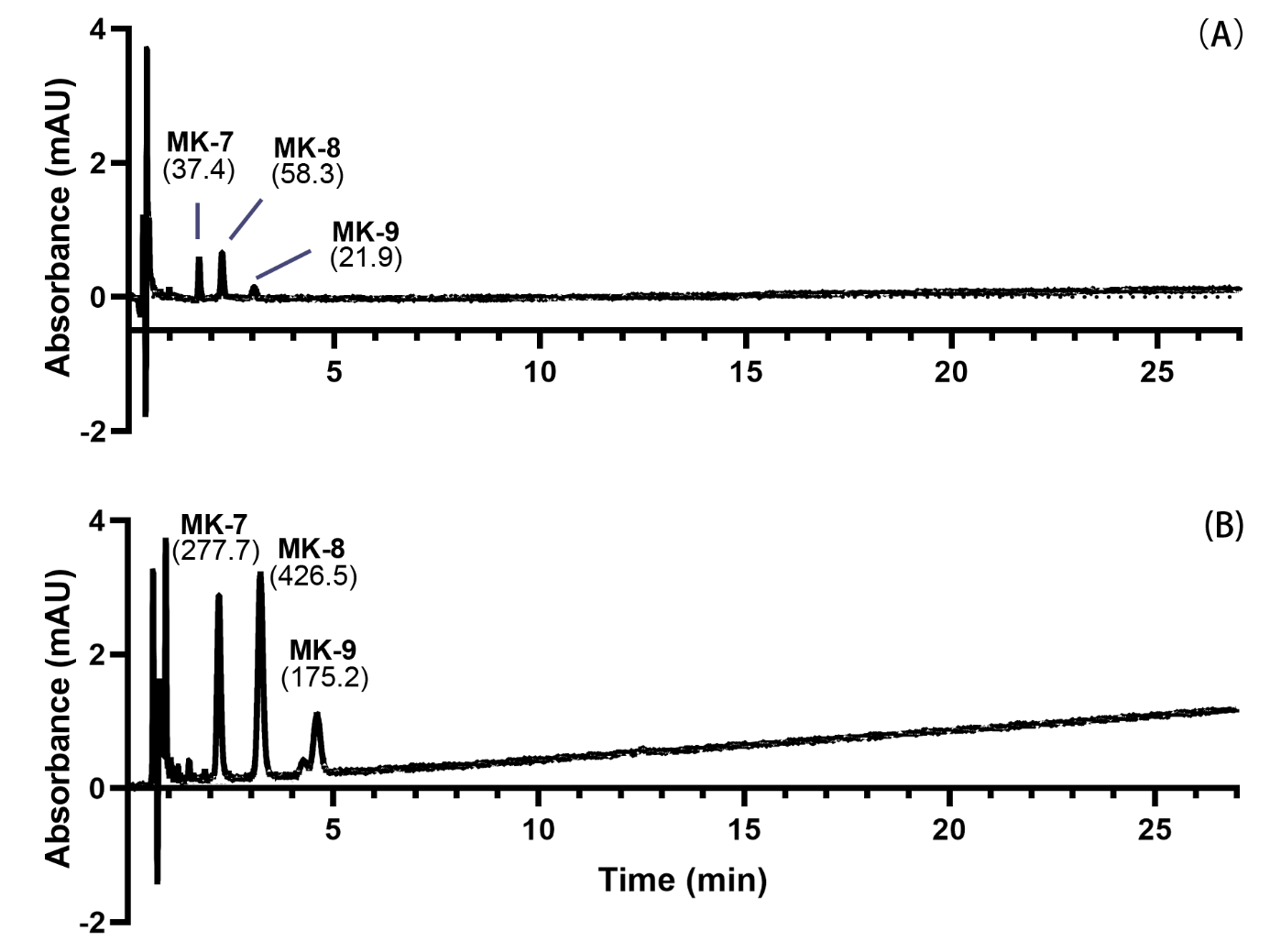
**Supplementary Figure 8.** UPLC-UV analysis of menaquinones (absorbance at 270 nm) extracted from type strain JCM 15475^T^. (A) and (B) Representative UPLC-UV analysis of menaquinones extracted with the Collins method and LCM method, respectively. The peak area is shown in parentheses.


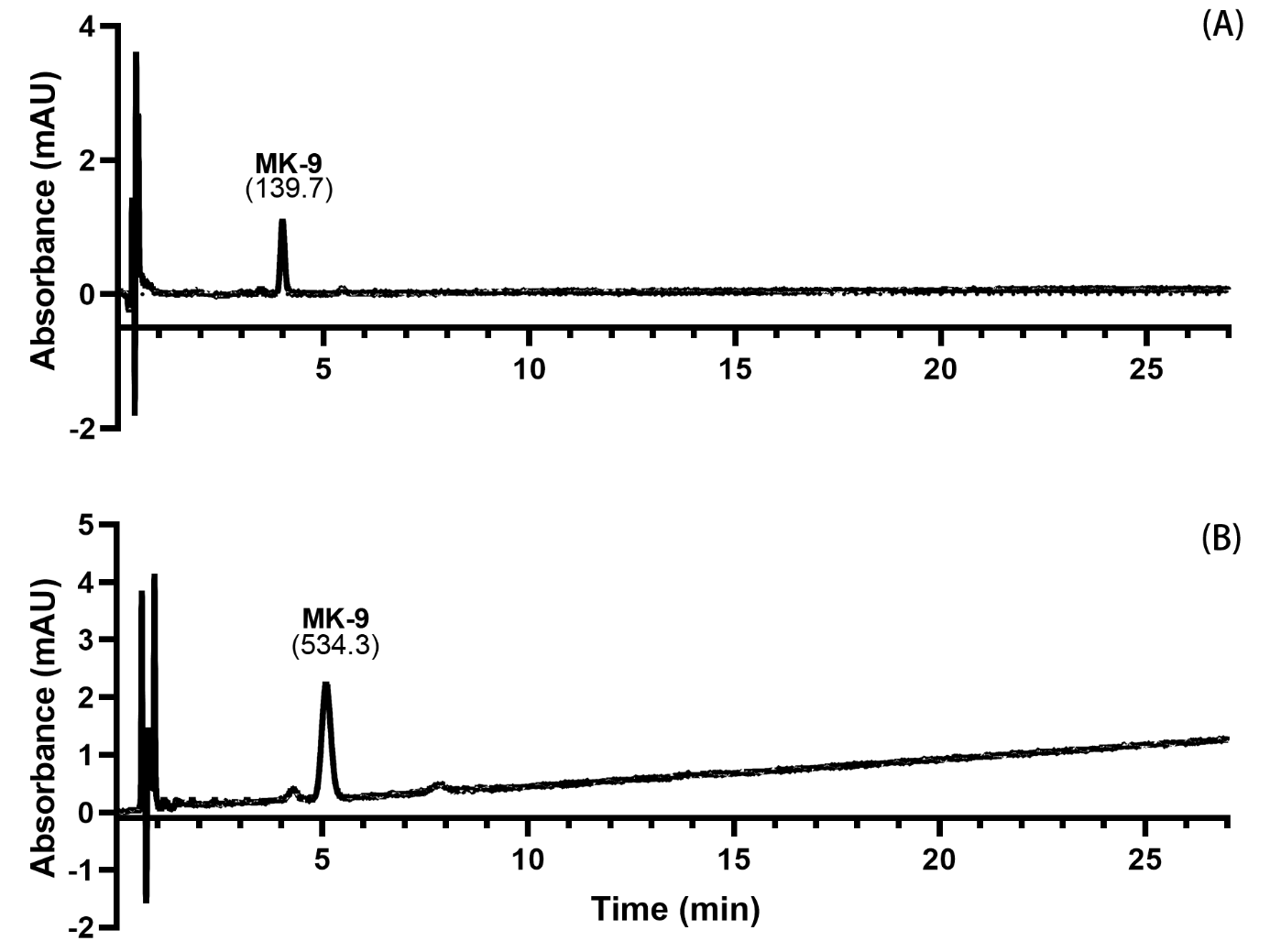
**Supplementary Figure 9.** UPLC-UV analysis of menaquinones (absorbance at 270 nm) extracted from type strain E2A^T^. (A) and (B) Representative UPLC-UV analysis of menaquinones extracted with the Collins method and LCM method, respectively. The peak area is shown in parentheses.


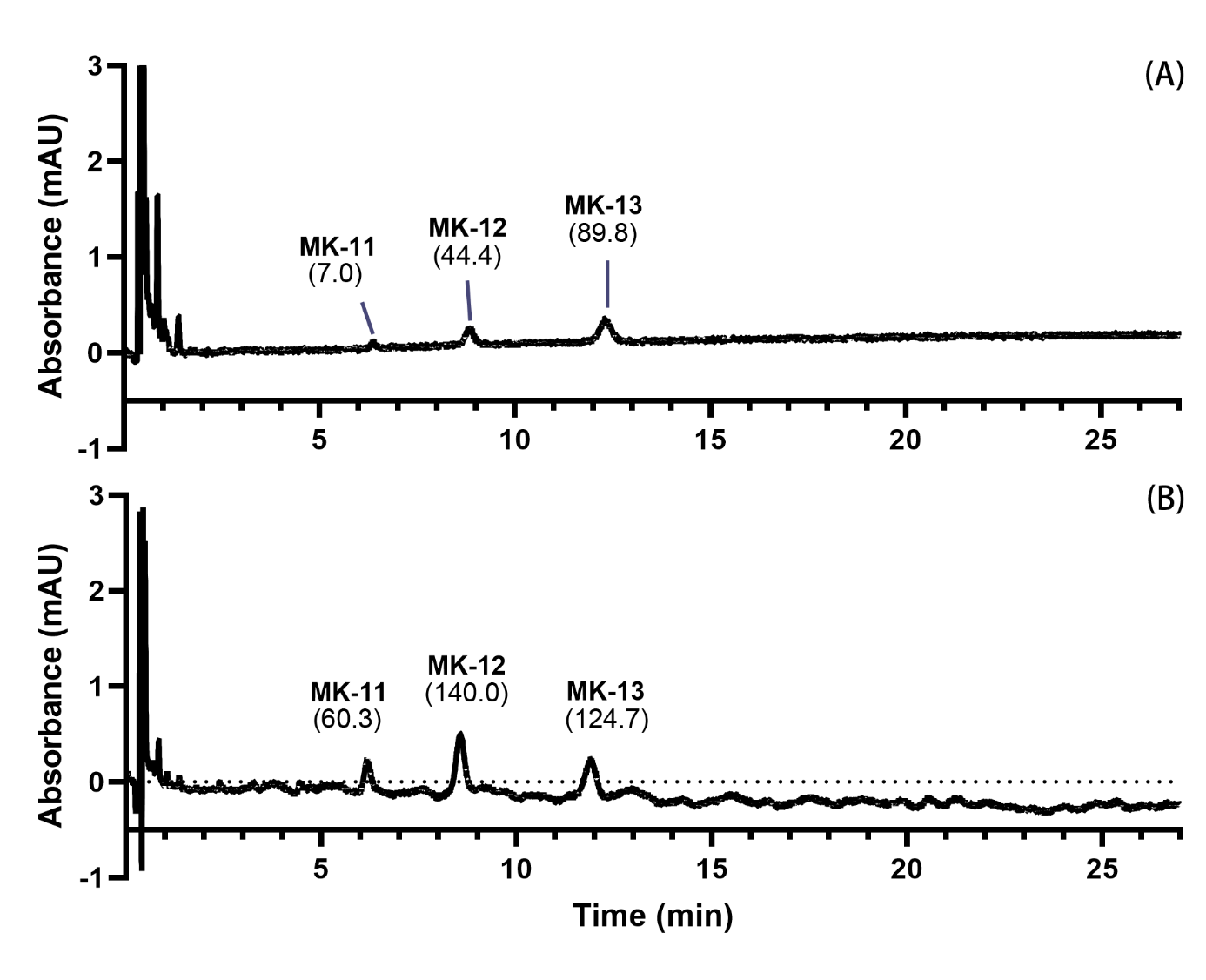
**Supplementary Figure 10.** UPLC-UV analysis of menaquinones (absorbance at 270 nm) extracted from type strain JCM 15138^T^. (A) and (B) Representative UPLC-UV analysis of menaquinones extracted with the Collins method and LCM method, respectively. The peak area is shown in parentheses.


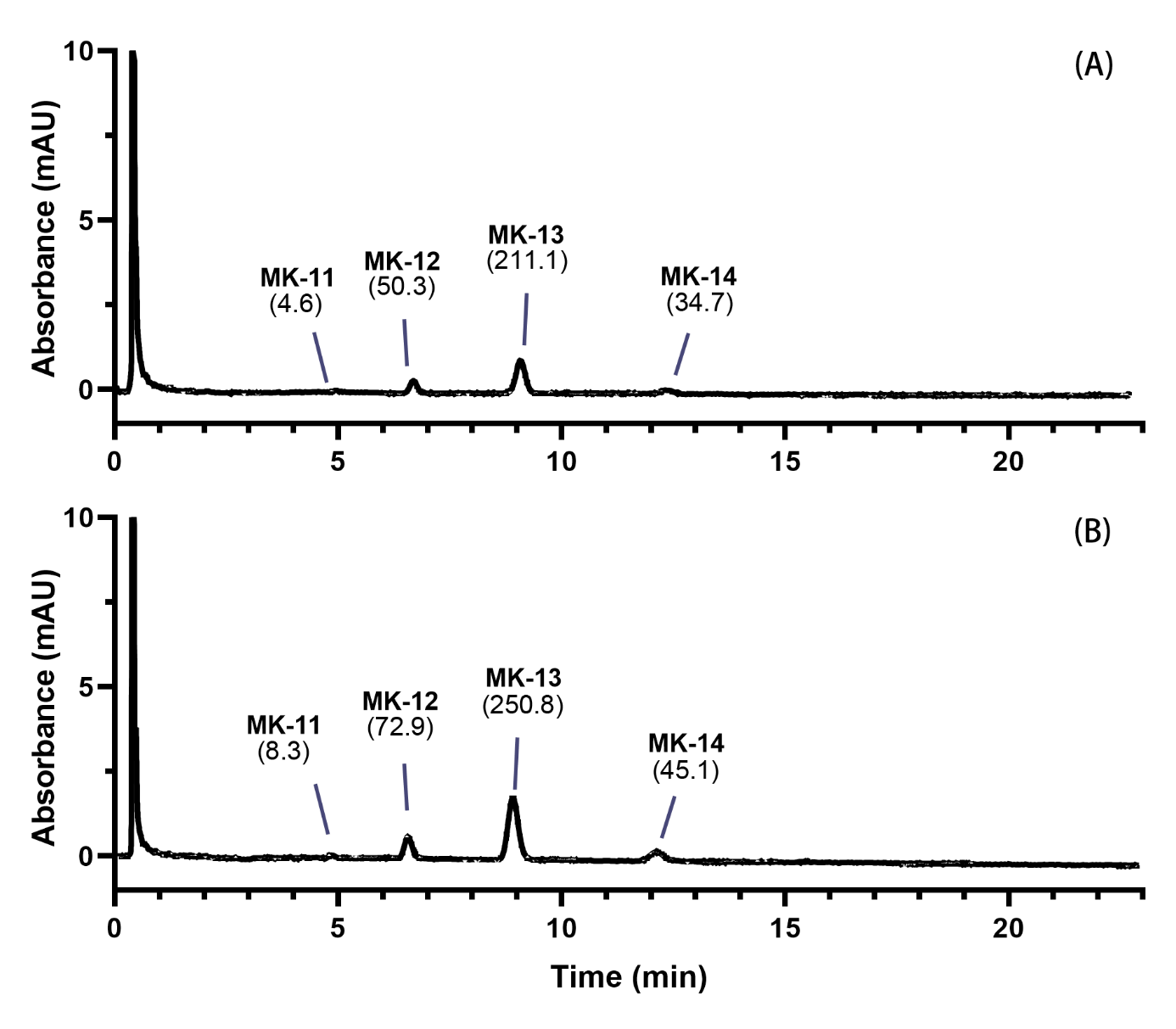


**Supplementary Figure 11.** UPLC-UV analysis of menaquinones (absorbance at 270 nm) extracted from type strain CCTCC AB 2016180^T^. (A) and (B) Representative UPLC-UV analysis of menaquinones extracted with the Collins method and LCM method, respectively. The peak area is shown in parentheses.
